# Survival prediction in peritoneal mesothelioma: a nomogram based on SEER data and a Chinese cohort

**DOI:** 10.3389/fendo.2024.1432787

**Published:** 2024-09-06

**Authors:** Yuting Fang, Midan Xiang, Zhichao Jiang, Hongrui Li, Guangwen Yuan, Wei Pei, Wenbin Li, Yongkun Sun

**Affiliations:** National Cancer Center/National Clinical Research Center for Cancer/Cancer Hospital, Chinese Academy of Medical Sciences and Peking Union Medical College, Beijing, China

**Keywords:** peritoneal mesothelioma, nomogram, SEER database, prognosis, risk stratification

## Abstract

**Objective:**

This study aimed to develop nomogram predicting overall survival (OS) of patients with peritoneal mesothelioma (PeM) using data from Surveillance, Epidemiology, and End Results (SEER) database and a Chinese institution.

**Methods:**

1,177 PeM patients from the SEER database were randomized into training and internal validation cohorts at a 7:3 ratio. An external validation cohort consisting of 109 patients was enrolled from a Chinese institution. Nomogram was constructed based on variables identified through multivariate Cox regression analysis and evaluated by consistency indices (C-index), calibration plots, and receiver operating characteristic (ROC) curves. Patients were stratified into different risk categories, and Kaplan-Meier survival analysis was used to assess OS differences among these groups.

**Results:**

The nomogram, incorporating age, gender, histological type, T stage, M stage, and surgical status, demonstrated strong predictive capability with C-index values of 0.669 for the training cohort, 0.668 for the internal validation cohort, and 0.646 for the external validation cohort. The nomogram effectively stratified patients into high-risk and low-risk groups, with the high-risk group exhibiting significantly poorer OS (P < 0.05). Multivariate analysis confirmed gender, age, surgical intervention, and M stage as independent prognostic factors (P < 0.05). Specifically, male gender, older age, and unspecified M stage were linked to worse outcomes, while surgical intervention was associated with improved survival.

**Conclusion:**

The nomogram provide a reliable tool for predicting the survival in PeM patients, facilitating more informed treatment decisions. Key independent prognostic factors include gender, age, surgical intervention, and M stage.

## Introduction

Mesothelioma is a lethal and aggressive disease that primarily affects the pleural and peritoneal membranes, often associated with asbestos exposure (1, 2). Peritoneal mesothelioma (PeM), accounting for 30% of all mesothelioma cases, is the second most common type after pleural mesothelioma (1). PeM typically presents with ascites, significant weight loss, fatigue, anorexia, a palpable abdominal mass, and symptoms indicative of intestinal obstruction (3). Diagnosis is frequently delayed due to the disease’s indolent progression and nonspecific clinical features, resulting in most cases being advanced at the time of suspicion.

For patients eligible for surgical intervention, cytoreductive surgery (CRS) combined with hyperthermic intraperitoneal chemotherapy (HIPEC) is the preferred treatment modality, demonstrating a median overall survival (OS) of 53 months and a five-year OS rate of 47% (4, 5). Conversely, patients not eligible for surgery typically receive systemic chemotherapy, although no established guidelines exist for PeM due to its rarity. Treatment strategies for these patients are often based on those for pleural mesothelioma, involving agents such as pemetrexed and platinum-based therapies, and may include targeted therapy or immunotherapy. An emerging treatment option is the dual-immunotherapy combination of PD-L1 and CTLA-4 inhibitors, such as ipilimumab plus nivolumab, which was approved by FDA in October 2020 for untreated pleural mesothelioma (6).

Developing advanced prognostic assessment methods for rare and highly malignant tumors is essential, particularly for diseases such as mesothelioma. These innovative assessment methods facilitate earlier prognosis prediction, enabling timely interventions and more precise treatment guidance. Currently, prognostic modeling for mesothelioma is predominantly focused on pleural mesothelioma, incorporating variables such as gender, age, histological type, surgical interventions, and chemotherapy (7, 8). However, prognostic models for PeM are notably lacking. The rarity of PeM challenges the establishment and validation of a reliable prognostic model, as only a limited number of retrospective studies have explored prognostic factors. These studies suggest that factors such as age, sex, CRS, HIPEC, the peritoneal cancer index, pathological type, and presence of distant metastases may influence prognosis (9, 10). Our study aims to identify risk factors and develop a predictive nomogram for PeM patients using data from both the SEER database and a Chinese institution.

## Methods

### Data source and study cohorts

Data for this study were obtained from the SEER*Stat database (version 8.4.3). Eligible participants were identified based on a diagnosis of neoplasms at sites coded C48.1 and C48.2, with histological types 9050/3, 9051/3, 9052/3, 9053/3, and 9055/3, recorded between the years 2004 and 2018. Patients without survival data or with survival durations less than 30 days were excluded. All included patients were restaged according to the AJCC eighth edition staging guidelines. The primary endpoint was overall survival (OS), defined as the duration from diagnosis to either death from any cause or the last follow-up.

Patients from the SEER database were randomized in a 7:3 ratio to form a training cohort and an internal validation cohort. The external validation cohort consisted of 109 PeM patients treated at the Cancer Hospital of the Chinese Academy of Medical Sciences and Peking Union Medical College from 2004 to 2022, with identical screening criteria to the SEER database. The last follow-up for the external validation cohort was in May 2023. The study was approved by the ethics committee of the Cancer Hospital, and informed consent was waived due to the retrospective nature of the study.

### Statistical analysis

Statistical analyses were conducted using RStudio(version 4.1.3). The chi-square test was applied to compare patient characteristics across the cohorts. Initial variables associated with survival was identified with univariate and multivariate Cox regression analyses. The variables were further refined via stepwise backward regression, selecting the model with the lowest Akaike Information Criterion (AIC) as the optimal model.

**Figure 1:**
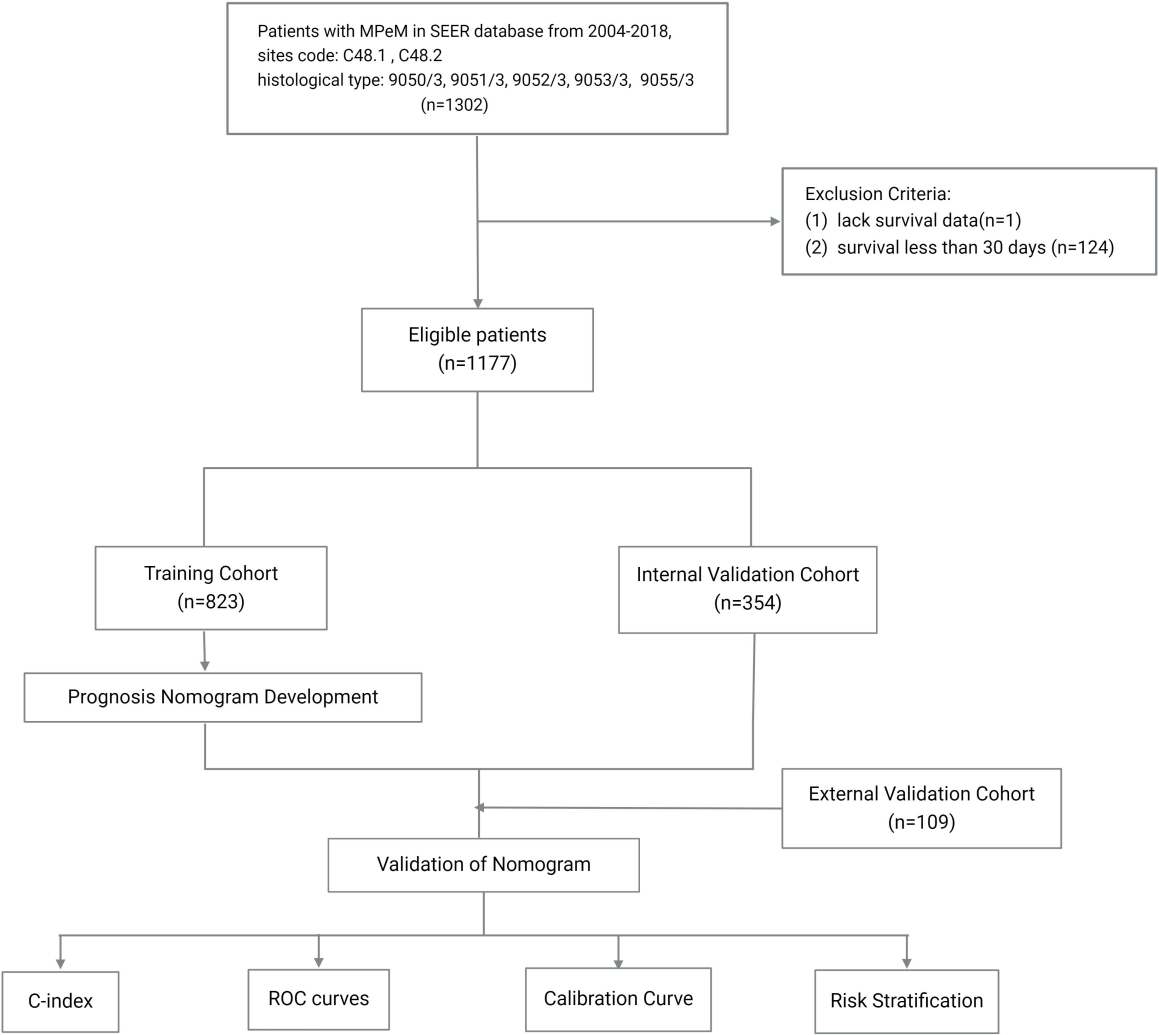
Flowchart detailing the patient screening process and study design.

Prognostic nomogram was developed to predict the OS rates of PeM patients at 0.5, 1, 2, and 5 years. Model performance was evaluated based on its discriminatory power and accuracy, assessed by the concordance index (C-index) and the area under the receiver operating characteristic (ROC) curve (AUC). Calibration curves were used to compare the predicted probabilities from the nomogram with the actual observed probabilities. Each patient’s relative risk, derived from the nomogram, was calculated, and the median relative risk score from the training cohort was used as a cutoff to stratify patients into different risk categories. Kaplan-Meier survival analysis was utilized to examine the differences in OS rates across these risk groups. A flowchart detailing the patient screening process and study design is presented in **Figure 1**.

## Results

### Characteristics of participants

A total of 1,177 individuals with PeM were identified in the SEER database and randomized into a training cohort (n = 823) and an internal validation cohort (n = 354), with no significant differences in baseline characteristics between the cohorts (P > 0.05). The external validation cohort from the Chinese institution included 109 Asian patients with PeM. Although significant differences were noted in most baseline variables compared to the training cohort (P < 0.05), the overall trends were similar. In the SEER database, the gender distribution was nearly balanced, with a female-to-male ratio of 1:1.18. The external cohort had a higher proportion of female patients, with a female-to-male ratio of 1.32:1. In the SEER database, the majority of patients (44.8%) were aged between 50 and 69 years, predominantly White (88.6%), with the largest proportion diagnosed between 2010 and 2016 (39.8%). Similarly, in the external validation cohort, most patients (64.2%) were aged between 50 and 69 years, with the most common period of diagnosis occurring from 2010 to 2016, accounting for 43.1%. Among those with known histological types in the SEER database, the epithelioid type was the most common, represented by 492 cases. In the external cohort, the epithelioid type was observed in 52.3% of patients. In the SEER cohort, a minority had known liver metastases (4.8%) and lung metastases (2.4%), while in the external cohort, there were 25 patients with liver metastases and 10 with lung metastases. Most patients in the SEER database had received chemotherapy (63.5%), fewer had undergone surgical treatments (45.4%), and radiotherapy was rare (1.1%). In the external cohort, a majority (83.5%) received chemotherapy, 62.4% had undergone surgical treatment, and only three patients received radiotherapy. Regarding staging in the SEER cohort, most patients had unclear TNM information, yet among those with known stages, T3, N0, and M0 were more prevalent, accounting for 10.9%, 27.0%, and 22.7%, respectively. In the external cohort, the T3, N0, and M1 stages were more prevalent, constituting 7.3%, 6.4%, and 60.6% of the cohort, respectively. All patients in the SEER cohort were restaged according to the AJCC eighth edition staging guidelines, with Stage III being the most common revised stage, involving 18.6% of the cohort. Similarly, Stage III was the most common stage in the external cohort, involving 63.3% of the patients (**Table 1**).

**Table 1:** Baseline characteristics of the training, internal validation and external validation cohort.

Characteristics	Train Cohort	Internal Validation Cohort	Overall	P value	External Validation Cohort	P value
	(N=823)	(N=354)	(N=1177)		(N=109)	
Sex				0.06		0.015
Female	362 (44.0%)	177 (50.0%)	539 (45.8%)		62 (56.9%)	
Male	461 (56.0%)	177 (50.0%)	638 (54.2%)		47 (43.1%)	
Age				0.28		<0.001
<50 years	175 (21.3%)	83 (23.4%)	258 (21.9%)		35 (32.1%)	
50-69 years	381 (46.3%)	146 (41.2%)	527 (44.8%)		70 (64.2%)	
>69 years	267 (32.4%)	125 (35.3%)	392 (33.3%)		4 (3.7%)	
Race				0.37		<0.001
Black	52 (6.3%)	15 (4.2%)	67 (5.7%)		0(0%)	
White	724 (88.0%)	319 (90.1%)	1043 (88.6%)		0 (0%)	
other	47 (5.7%)	20 (5.6%)	67 (5.7%)		109 (100%)	
Diagnose_date				0.71		<0.001
before 2010	317 (38.5%)	132 (37.3%)	449 (38.1%)		20 (18.3%)	
2010-2016	321 (39.0%)	147 (41.5%)	468 (39.8%)		47 (43.1%)	
after 2016	185 (22.5%)	75 (21.2%)	260 (22.1%)		42 (38.5%)	
Chemotherapy				0.37		<0.001
Yes	515 (62.6%)	232 (65.5%)	747 (63.5%)		91(83.5%)	
No/Unknown	308 (37.4%)	122 (34.5%)	430 (36.5%)		18(16.5%)	
Surgery				0.79		0.001
Yes	376 (45.7%)	158 (44.6%)	534 (45.4%)		68 (62.4%)	
No	447 (54.3%)	196 (55.4%)	643 (54.6%)		41 (37.6%)	
Radiation				0.72		0.25
Yes	8 (1.0%)	5 (1.4%)	13 (1.1%)		3 (2.8%)	
No	815 (99.0%)	349 (98.6%)	1164 (98.9%)		106 (97.2%)	
Livermeta				0.49		<0.001
Yes	37 (4.5%)	20 (5.6%)	57 (4.8%)		25 (22.9%)	
No/Unknown	786 (95.5%)	334 (94.4%)	1120 (95.2%)		84 (77.1%)	
Lungmeta				0.09		<0.001
Yes	15 (1.8%)	13 (3.7%)	28 (2.4%)		10 (9.2%)	
No/Unknown	808 (98.2%)	341 (96.3%)	1149 (97.6%)		99 (90.8%)	
Histology				0.27		0.017
Biphasic	33 (4.0%)	10 (2.8%)	43 (3.7%)		8 (7.3%)	
Epithelioid	330 (40.1%)	162 (45.8%)	492 (41.8%)		57 (52.3%)	
Fibrous	24 (2.9%)	11 (3.1%)	35 (3.0%)		2 (1.8%)	
unknown	436 (53.0%)	171 (48.3%)	607 (51.6%)		42 (38.5%)	
T stage				0.02		0.62
T1	17 (2.1%)	4 (1.1%)	21 (1.8%)		2 (1.8%)	
T2	33 (4.0%)	15 (4.2%)	48 (4.1%)		2 (1.8%)	
T3	75 (9.1%)	53 (15.0%)	128 (10.9%)		8 (7.3%)	
Tx	698 (84.8%)	282 (79.7%)	980 (83.3%)		97 (89.0%)	
N stage				0.58		<0.001
N0	220 (26.7%)	98 (27.7%)	318 (27.0%)		7 (6.4%)	
N1	30 (3.6%)	17 (4.8%)	47 (4.0%)		5 (4.6%)	
Nx	573 (69.6%)	239 (67.5%)	812 (69.0%)		97 (89.0%)	
M stage				0.61		<0.001
M0	186 (22.6%)	81 (22.9%)	267 (22.7%)		43 (39.4%)	
M1	135 (16.4%)	66 (18.6%)	201 (17.1%)		66 (60.6%)	
Unknown	502 (61.0%)	207 (58.5%)	709 (60.2%)		0 (0%)	
Stage				0.72		<0.001
I	9 (1.1%)	3 (0.8%)	12 (1.0%)		0 (0%)	
II	57 (6.9%)	28 (7.9%)	85 (7.2%)		3 (2.8%)	
III	148 (18.0%)	71 (20.1%)	219 (18.6%)		69 (63.3%)	
Unknown	609 (74.0%)	252 (71.2%)	861 (73.2%)		37 (33.9%)	

### Identification of independent prognostic factors for model development

**Figure 2:**
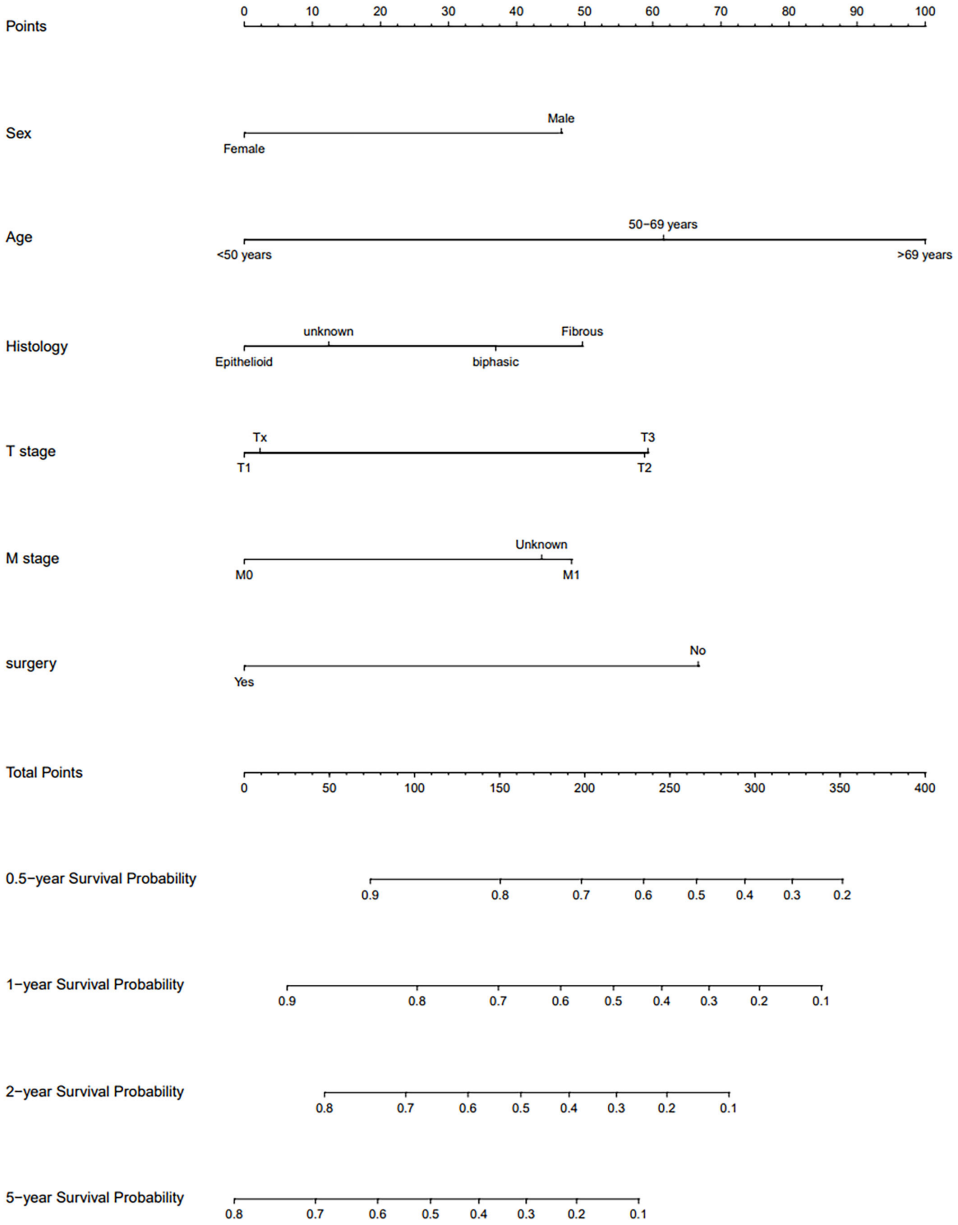
Nomogram for predicting 0.5, 1, 2 and 5 year OS of PeM patients.

Univariate Cox regression analysis conducted within the training cohort identified age, gender, surgery, radiotherapy, lung metastasis, T stage, and M stage as variables significantly correlated with OS(P < 0.05). Variables with a P-value less than 0.1 from the univariate analysis were subsequently incorporated into the multivariate Cox regression analysis. This analysis revealed that gender, age, surgery, and M stage were independent prognostic factors(P<0.05). Specifically, being male was associated with a worse prognosis (Hazard Ratio [HR]: 1.57, 95% Confidence Interval [CI]: 1.30-1.91, P < 0.001). Patients aged 50-69 years (HR: 1.82, 95% CI: 1.41-2.35, P < 0.001) and those over 69 years (HR: 2.68, 95% CI: 2.05-3.49, P < 0.001) had worse outcomes compared to patients younger than 50 years. Patients who underwent surgical treatment exhibited a better prognosis (HR: 0.52, 95% CI: 0.43-0.62, P < 0.001). Additionally, patients with unspecified M stage had a poorer prognosis compared to those clearly staged as M0 (HR: 1.52, 95% CI: 1.15-2.01, P=0.003). Considering clinical relevance, additional variables including histological type, diagnosis time, T stage, N stage, and overall stage were also included in the variable selection pool. The optimal model was established using stepwise backward regression based on the lowest AIC values (**Figure 2**). The variables incorporated into the final prognostic model include age, gender, histological type, T stage, M stage, and surgery. The outcomes of the Cox regression survival analysis based on OS are presented in **Table 2**.

**Table 2:** Selection of variables associated with OS by univariate and multivariate Cox proportional hazards analysis in the training cohort.

Characteristics	Univariate analysis		Multivariate analysis	
	HR(95%CI)	P Value	HR(95%CI)	P Value
Sex				
Female	reference		reference	
Male	1.58(1.33-1.87)	<0.001	1.57(1.30-1.91)	<0.001
Age				
<50 years	reference		reference	
50-69 years	1.97(1.53-2.53)	<0.001	1.82(1.41-2.35)	<0.001
>69 years	3.14(2.43-4.07)	<0.001	2.68(2.05-3.49)	<0.001
Race				
Black	reference		reference	
White	1.39(0.95-2.03)	0.09	1.08(0.73-1.61)	0.7
other	1.43(0.88-2.51)	0.14	1.22(0.71-2.09)	0.47
Diagnose year				
2010-2016	reference			
after 2016	0.85(0.65-1.12)	0.25		
before 2010	1.09(0.91-1.30)	0.35		
Chemotherapy				
No/Unknown	reference			
Yes	0.96(0.81-1.13)	0.61		
Surgery				
No	reference		reference	
Yes	0.47(0.40-0.56)	<0.001	0.52(0.43-0.62)	<0.001
Radiation				
No	reference		reference	
Yes	2.15(1.07-4.33)	0.03	1.77(0.86-3.65)	0.2
Livermeta				
No/Unknown	reference		reference	
Yes	1.47(0.99-2.21)	0.06	1.23(0.81-1.88)	0.34
Lungmeta				
No/Unknown	reference		reference	
Yes	1.79(1.01-3.17)	0.047	1.54(0.83-2.83)	0.17
Histology				
biphasic	reference			
Epithelioid	0.80(0.51-1.26)	0.34		
Fibrous	1.47(0.79-2.73)	0.22		
unknown	0.97(0.62-1.51)	0.89		
T stage				
T1	reference		reference	
T2	2.24(1.11-4.51)	0.02	1.73(0.67-4.44)	0.26
T3	1.29(0.68-2.46)	0.44	1.66(0.66-4.21)	0.28
Tx	1.41(0.77-2.56)	0.26	1.02(0.43-2.40)	0.96
N stage				
N0	reference			
N1	0.83(0.52-1.33)	0.44		
Nx	1.00(0.83-1.21)	0.98		
stageM				
M0	reference		reference	
M1	1.83(1.41-2.37)	<0.001	1.08(0.51-2.29)	0.84
Unknown	1.32(1.07-1.63)	0.01	1.52(1.15-2.01)	0.003
Stage				
I	reference		reference	
II	1.47(0.58-3.71)	0.42	1.42(0.39-5.17)	0.59
III	2.20(0.90-5.38)	0.08	1.79(0.48-6.65)	0.38
Unknown	1.57(0.65-3.80)	0.31	1.27(0.37-4.41)	0.71

### Creation and validation of nomogram

A nomogram was developed to predict long-term survival in patients with PeM using six key variables. By summing the scores assigned to these variables, we estimated the OS rates at 0.5, 1, 2, and 5 years. The accuracy of the model was validated using the C-index, ROC curves, and calibration curves. The C-index were 0.669 (95% CI, 0.645-0.693) for the training cohort, 0.668 (95% CI, 0.631-0.705) for the internal validation cohort, and 0.646 (95% CI, 0.575-0.717) for the external validation cohort.

**Figure 3:**
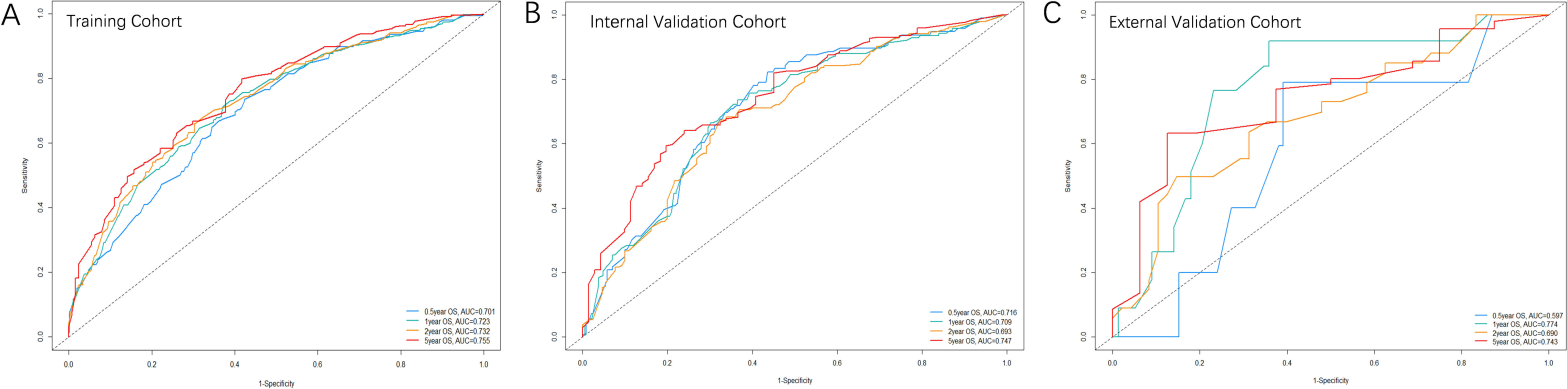
The ROC curves of the nomogram predicting OS rate in the three cohorts: **(A)** Training cohort; **(B)** Internal validation cohort; **(C)** External validation cohort.

**Figure 3** illustrates the area under the curve (AUC) values of the nomogram predicting 0.5-, 1-, 2-, and 5-year OS rate across the three cohorts. For the training cohort, the AUC values were 0.701 (95% CI, 0.660-0.742) at 0.5 years, 0.723 (95% CI, 0.686-0.759) at 1 year, 0.732 (95% CI, 0.695-0.769) at 2 years, and 0.755 (95% CI, 0.709-0.800) at 5 years. In the internal validation cohort, the values were 0.717 (95% CI, 0.658-0.776) at 0.5 years, 0.709 (95% CI, 0.652-0.756) at 1 year, 0.693 (95% CI, 0.634-0.752) at 2 years, and 0.747 (95% CI, 0.683-0.811) at 5 years. For the external validation cohort, the AUC values were 0.600 (95% CI, 0.371-0.823) at 0.5 years, 0.774 (95% CI, 0.643-0.904) at 1 year, 0.690 (95% CI, 0.573-0.807) at 2 years, and 0.743 (95% CI, 0.612-0.874) at 5 years, respectively.

**Figure 4:**
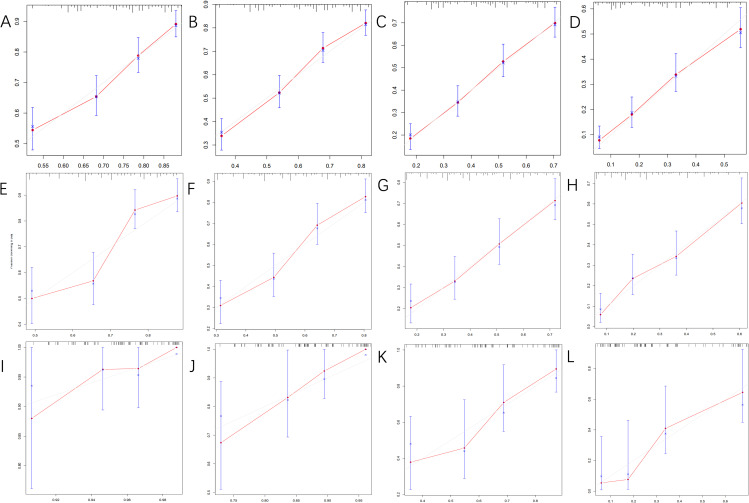
The calibration curves for predicting OS at **(A)** 0.5-year and **(B)** 1-year and **(C)** 2-year and **(D)** 5-year in the training cohort, and at **(E)** 0.5-year **(F)** 1-year and **(G)** 2-year and **(H)** 5-year in the internal validation cohort, and at **(I)** 0.5-year **(J)** 1-year and **(K)** 2-year and **(L)** 5-year in the external validation cohort.

**Figure 4** displays the calibration curves for the prediction model, which compare the actual OS rates at 0.5, 1, 2, and 5 years with the predicted probabilities across the three cohorts. These curves reveal a high level of agreement between the survival rates predicted by the nomograms and the observed rates in the actual patient population, indicating good consistency.

### Risk stratification based on model predictions

**Figure 5:**
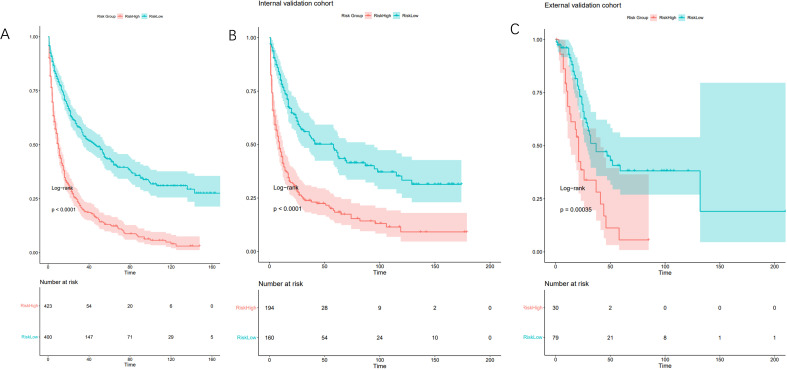
Kaplan-Meier curves for correlation with OS for the low and high- risk groups in the training cohort **(A)** (p<0.001), internal validation cohort **(B)** (p<0.001) and external validation cohort **(C)** (p<0.001).

Relative risk was assessed based on the model, and patients in the training and validation cohorts were categorized into high-risk (relative risk ≥ 2.00) and low-risk (relative risk < 2.00) groups using the median relative risk value (cut-off=2.00) from the training cohort. Kaplan-Meier survival analysis demonstrated significant differences in OS between these risk groups (**Figure 5**). The P-values for the training, internal validation, and external validation cohorts were all less than 0.001, indicating that the nomogram effectively stratifies risk among patients with PeM.

## Discussion

PeM is characterized by its low incidence rate, presenting significant challenges for researchers in gathering adequate case data from single-center studies. The SEER database, as the primary cancer statistics repository in the United States, offers a comprehensive and diverse sample of U.S. patients spanning various years, making it an exceptionally valuable resource for studying rare tumors. The extensive data available through SEER facilitates robust research by offering a broader geographical and temporal range, thereby enhancing the statistical power and validity of findings in rare cancer research.

Statistically, there were no significant differences in baseline characteristics between the training and the internal validation cohorts (P > 0.05). Due to the rarity of the disease, the collection period was extended from 2004 to 2022 to include a larger number of cases for the external validation cohort. Although significant differences were observed in most baseline characteristics between the training and the external validation cohorts (P < 0.05), the overall trends were almost similar. Despite these differences, the ROC curves, AUC values, and C-index all confirmed that the prognostic model developed using the SEER database performed well in the external validation cohort. The model effectively differentiated survival outcomes among various risk groups, underscoring its applicability and robustness in external cohorts.

Univariate analysis on the training cohort identified age, gender, histology type, surgery, radiation, lung metastasis, T stage, and M stage as factors significantly associated with OS(P < 0.05). Further multivariate Cox regression analysis determined that age, gender, surgery, and M stage are independent prognostic factors for PeM.

Considering clinical relevance, despite some variables such as histological type and AJCC staging showing no significant correlation with OS in both univariate and multivariate analyses, they were retained in the variable selection pool. Using backward stepwise selection, six variables were chosen for the final model: age, gender, histological type, surgery, T stage, and M stage. The model’s discriminatory power and accuracy were subsequently validated through the C-index, AUC values, and calibration curves, confirming the robustness of the prognostic model developed.

Age was incorporated as a categorical variable in this prognostic model. Both univariate and multivariate analyses demonstrated that patients younger than 50 years exhibited a more favorable prognosis. This may be attributed to younger patients generally having better overall health condition and a higher tolerance for treatment. Further studies have shown that mesothelioma patients with a family history of mesothelioma or other cancers, or those with early-onset mesothelioma (diagnosed before age 50), are more likely to carry inherited germline mutations. Patients with such mutations often have a better prognosis (11, 12), potentially due to increased sensitivity to platinum-based chemotherapy, facilitated by loss-of-function mutations in DNA repair genes (13).

Gender is also a significant variable in this prognostic model. Our analyses suggest that female patients generally have a better prognosis than male patients, consistent with previous findings (9). The prognostic differences between genders may be influenced by hormonal factors in the disease’s pathobiology. Pinton et al. demonstrated that the presence of estrogen receptor β (ER-β) in tumors is indicative of a better prognosis (14). Additionally, a case report detailing the seven-year survival of a female patient with PeM further underscores the potential influence of ER-β positivity on her prolonged survival (15).

Histological type is a crucial component of this prognostic model, with previous studies consistently identifying it as a prognostic factor. Specifically, the epithelioid type is known to have the best prognosis (9, 16, 17). However, in our study, a significant number of patients in the SEER database were categorized as “Unknown” for histological type, and only 10 cases in the external validation cohort were non-epithelioid, which may explain the lack of significant correlation found between histological type and OS in our study. Epithelioid mesothelioma is typically associated with a better prognosis, possibly due to its lower aggressiveness and slower growth rate compared to other types, such as sarcomatoid or biphasic (18). Furthermore, studies have shown that patients with the epithelioid subtype often respond better to CRS and HIPEC, resulting in improved OS rates post-surgery (4, 19, 20).

Surgical intervention is a significant component of this prognostic model. Multivariate Cox regression analysis in our study identified whether or not surgery was performed as an independent prognostic factor, with surgical patients demonstrating better outcomes, consistent with previous findings. Patients treated with CRS and HIPEC showed an extended median OS of up to 53 months, and a five-year OS rate of approximately 47% (4). Unfortunately, the majority of patients with advanced disease do not have the opportunity for surgical intervention, highlighting the critical importance of early detection and diagnosis for enhancing survival outcomes. Future research may explore ways to refine surgical techniques and enhance the integration with HIPEC, including optimizing the selection of chemotherapeutic agents used in HIPEC.

For patients with inoperable PeM, systemic chemotherapy remains a primary treatment approach, typically employing regimens adapted from those used in pleural mesothelioma, such as pemetrexed plus cisplatin combined with targeted or immunotherapy. Research has shown that some regimens can notably extend OS. For instance, a multicenter Phase III clinical trial involving 456 patients with pleural mesothelioma showed that those receiving pemetrexed plus cisplatin had a median OS of 12.1 months compared to 9.3 months for those receiving cisplatin alone (P=0.020), with partial response (PR) rates of 41.3% versus 16.7% (P<0.001) (21). Another Phase III randomized controlled trial with 448 pleural mesothelioma patients found that combining bevacizumab with pemetrexed and cisplatin significantly increased median OS from 16.1 months to 18.8 months (P=0.0167) (22). However, in our study, chemotherapy was not identified as a prognostic factor and was therefore excluded from the final model. Further exploratory Cox analysis on the inoperable patient cohort revealed no significant relationship between chemotherapy and prognosis (P=0.70). This may be due to the SEER database’s lack of detailed information on specific chemotherapy regimens, which could obscure the effectiveness of certain treatments, particularly in the context of recent advances in immunotherapy and targeted therapies, such as inhibitors of VEGFR and PD-1 (6, 23, 24). The absence of detailed information on targeted and immune therapies represents a limitation in our model construction.

This study presents the first prognostic model focused on PeM, with external validation in a cohort of 109 PeM patients from Asia demonstrating its good applicability of the predictive model in real-world settings. However, as a retrospective analysis based on a public database, this study has several limitations. While the SEER database offers comprehensive details on treatments such as chemotherapy, radiotherapy, and surgery, it lacks specific information on chemotherapy regimens, targeted therapies, and immunotherapy. Given the promising clinical outcomes of these advanced treatments in mesothelioma, the absence of this data limits a thorough prognostic analysis. Furthermore, the database does not include certain symptomatic, diagnostic, and personal history information, such as the presence of ascites, tumor marker levels, asbestos exposure, or socioeconomic factors, which have been shown to correlate with prognosis in previous studies. This lack of data hinders the development of a more comprehensive prognostic model. Additionally, many variables, such as histological type and TNM staging information, are often recorded as “Unknown”, complicating the construction of an effective prognostic model and potentially affecting its accuracy and clinical utility. Moreover, this study faces further limitations due to the heterogeneity in ethnic representation across the training and validation cohorts. While the SEER database encompasses various ethnic groups, our external validation cohort is comprised solely of Asian patients. Although the model was validated effectively within this cohort, indicating satisfactory applicability across diverse populations, future research should aim to increase sample sizes and include a broader range of ethnicities to enhance the model’s effectiveness and applicability across different demographic groups. Additionally, collaborating with registries that provide detailed information on chemotherapy regimens, targeted therapies, immunotherapy, and other relevant clinical and personal history data will be crucial for developing more comprehensive prognostic models.

## Summary

In summary, we successfully developed and validated a nomogram for predicting the OS of PeM patients, offering a more accurate foundation for treatment decisions. This represents the first prognostic model specifically for PeM that leverages data not only from the SEER database but also includes external validation with an Asian cohort. This dual-source approach has demonstrated that the predictive model retains strong applicability for patients in real-world settings across Asia. Additionally, our analysis of the SEER database identified age, gender, surgical intervention, and M stage as independent prognostic factors for PeM patients. This study makes a significant contribution to the field by offering a tailored prognostic tool that enhances the understanding of PeM and supports targeted clinical decision-making.

## Data Availability

The raw data supporting the conclusions of this article will be made available by the authors, without undue reservation.

## References

[1] Greenbaum A Alexander HR. Peritoneal mesothelioma. Transl Lung Cancer Res. (2020) 9:S120–S32. doi: 10.21037/tlcr PMC7082256 32206577

[2] Pouliquen DL Kopecka J. Malignant mesothelioma. Cancers (Basel). (2021) 13:1–3. doi: 10.3390/cancers13143447 PMC8307269 34298661

[3] García-Fadrique A Mehta A Mohamed F Dayal S Cecil T Moran BJ. Clinical presentation, diagnosis, classification and management of peritoneal mesothelioma: a review. J Gastrointest Oncol. (2017) 8:915–24. doi: 10.21037/jgo PMC5674249 29184697

[4] Yan TD Deraco M Baratti D Kusamura S Elias D Glehen O. Cytoreductive surgery and hyperthermic intraperitoneal chemotherapy for Malignant peritoneal mesothelioma: multi-institutional experience. J Clin Oncol. (2009) 27:6237–42. doi: 10.1200/JCO.2009.23.9640 19917862

[5] Feldman AL Libutti SK Pingpank JF Bartlett DL Beresnev TH Mavroukakis SM. Analysis of factors associated with outcome in patients with Malignant peritoneal mesothelioma undergoing surgical debulking and intraperitoneal chemotherapy. J Clin Oncol. (2003) 21:4560–7. doi: 10.1200/JCO.2003.04.150 14673042

[6] Baas P Scherpereel A Nowak AK Fujimoto N Peters S Tsao AS. First-line nivolumab plus ipilimumab in unresectable Malignant pleural mesothelioma (CheckMate 743): a multicentre, randomised, open-label, phase 3 trial. Lancet. (2021) 397:375–86. doi: 10.1016/S0140-6736(20)32714-8 33485464

[7] Chen S Yu W Shao S Xiao J Bai H Pu Y. Establishment of predictive nomogram and web-based survival risk calculator for Malignant pleural mesothelioma: A SEER database analysis. Front Oncol. (2022) 12:1027149. doi: 10.3389/fonc.2022.1027149 36276110 PMC9585232

[8] Zhang X Chang L Zhu Y Mao Y Zhang T Zhang Q. Establishment and validation of nomograms to predict survival probability of advanced Malignant pleural mesothelioma based on the SEER database and a Chinese medical institution. Front Endocrinol (Lausanne). (2023) 14:1139222. doi: 10.3389/fendo.2023.1139222 37124752 PMC10140559

[9] Yonemura Y Canbay E Wakama S Sako S Ishibashi H Hirano M. Prognostic factors of Malignant peritoneal mesothelioma experienced in Japanese peritoneal metastasis center. Gan To Kagaku Ryoho. (2019) 46:395–9. 30914572

[10] Yin W Zheng G Yang K Song H Liang Y. Analysis of prognostic factors of patients with Malignant peritoneal mesothelioma. World J Surg Oncol. (2018) 16:44. doi: 10.1186/s12957-018-1350-5 29506546 PMC5836427

[11] Baumann F Flores E Napolitano A Kanodia S Taioli E Pass H. Mesothelioma patients with germline BAP1 mutations have 7-fold improved long-term survival. Carcinogenesis. (2015) 36:76–81. doi: 10.1093/carcin/bgu227 25380601 PMC4291047

[12] Pastorino S Yoshikawa Y Pass HI Emi M Nasu M Pagano I. A subset of mesotheliomas with improved survival occurring in carriers of BAP1 and other germline mutations. J Clin Oncol. (2018) 36:JCO2018790352. doi: 10.1200/JCO.2018.79.0352 30376426 PMC7162737

[13] Hassan R Morrow B Thomas A Walsh T Lee MK Gulsuner S. Inherited predisposition to Malignant mesothelioma and overall survival following platinum chemotherapy. Proc Natl Acad Sci U S A. (2019) 116:9008–13. doi: 10.1073/pnas.1821510116 PMC6500142 30975761

[14] Pinton G Brunelli E Murer B Puntoni R Puntoni M Fennell DA. Estrogen receptor-beta affects the prognosis of human Malignant mesothelioma. Cancer Res. (2009) 69:4598–604. doi: 10.1158/0008-5472.CAN-08-4523 19487281

[15] Pillai K Akhter J Pourgholami MH Morris DL. Peritoneal mesothelioma in a woman who has survived for seven years: a case report. J Med Case Rep. (2011) 5:36. doi: 10.1186/1752-1947-5-36 21269462 PMC3042400

[16] Calthorpe L Romero-Hernandez F Miller P Conroy PC Hirose K Kim A. Contemporary trends in Malignant peritoneal mesothelioma: incidence and survival in the United States. Cancers (Basel). (2022) 15:1–12. doi: 10.3390/cancers15010229 36612225 PMC9818958

[17] Ma J Zhang S. Prognostic factors of Malignant peritoneal mesothelioma: a retrospective study of 52 female patients. World J Surg Oncol. (2022) 20:219. doi: 10.1186/s12957-022-02688-x 35765009 PMC9241280

[18] Liu S Staats P Lee M Alexander HR Burke AP. Diffuse mesothelioma of the peritoneum: correlation between histological and clinical parameters and survival in 73 patients. Pathology. (2014) 46:604–9. doi: 10.1097/PAT.0000000000000181 25393250

[19] Sugarbaker PH Turaga KK Alexander HR Deraco M Hesdorffer M. Management of Malignant peritoneal mesothelioma using cytoreductive surgery and perioperative chemotherapy. J Oncol Pract. (2016) 12:928–35. doi: 10.1200/JOP.2016.011908 27858561

[20] Lee M Alexander HR Burke A. Diffuse mesothelioma of the peritoneum: a pathological study of 64 tumours treated with cytoreductive therapy. Pathology. (2013) 45:464–73. doi: 10.1097/PAT.0b013e3283631cce 23846294

[21] Vogelzang NJ Rusthoven JJ Symanowski J Denham C Kaukel E Ruffie P. Phase III study of pemetrexed in combination with cisplatin versus cisplatin alone in patients with Malignant pleural mesothelioma. J Clin Oncol. (2003) 21:2636–44. doi: 10.1200/JCO.2003.11.136 12860938

[22] Zalcman G Mazieres J Margery J Greillier L Audigier-Valette C Moro-Sibilot D. Bevacizumab for newly diagnosed pleural mesothelioma in the Mesothelioma Avastin Cisplatin Pemetrexed Study (MAPS): a randomised, controlled, open-label, phase 3 trial. Lancet. (2016) 387:1405–14. doi: 10.1016/S0140-6736(15)01238-6 26719230

[23] Fennell DA Ewings S Ottensmeier C Califano R Hanna GG Hill K. Nivolumab versus placebo in patients with relapsed Malignant mesothelioma (CONFIRM): a multicentre, double-blind, randomised, phase 3 trial. Lancet Oncol. (2021) 22:1530–40. doi: 10.1016/S1470-2045(21)00471-X PMC8560642 34656227

[24] Scherpereel A Mazieres J Greillier L Lantuejoul S Dô P Bylicki O. Nivolumab or nivolumab plus ipilimumab in patients with relapsed Malignant pleural mesothelioma (IFCT-1501 MAPS2): a multicentre, open-label, randomised, non-comparative, phase 2 trial. Lancet Oncol. (2019) 20:239–53. doi: 10.1016/S1470-2045(18)30765-4 30660609

